# ^18^F-fluorodeoxyglucose positron emission tomography/computed tomography findings of gastric lymphoma: Comparisons with gastric cancer

**DOI:** 10.3892/ol.2014.2412

**Published:** 2014-08-04

**Authors:** JIANG WU, HONG ZHU, KAI LI, XIN-GANG WANG, YI GUI, GUANG-MING LU

**Affiliations:** 1 Department of Nuclear Medicine, Jinling Hospital, School of Medicine, Nanjing University, Nanjing, Jiangsu 210002, P.R. China; 2 Department of Pharmacology, Soochow University, Suzhou, Jiangsu 215123, P.R. China; 3 Department of Medical Imaging, Jinling Hospital, School of Medicine, Nanjing University, Nanjing, Jiangsu 210002, P.R. China

**Keywords:** positron emission tomography, computed tomography, gastric lymphoma, gastric cancer

## Abstract

The role of ^18^F-fluorodeoxyglucose positron emission tomography/computed tomography (^18^F-FDG PET/CT) in numerous malignant tumors, including gastric lymphoma, is well-established. However, there have been few studies with regard to the ^18^F-FDG PET/CT features of gastric lymphoma. The aim of the present study was to characterize the ^18^F-FDG PET/CT features of gastric lymphoma, which were compared with those of gastric cancer. Prior to treatment, ^18^F-FDG PET/CT was performed on 24 patients with gastric lymphoma and 43 patients with gastric cancer. The ^18^F-FDG PET/CT pattern of gastric wall lesions was classified as one of three types: Type I, diffuse thickening of the gastric wall with increased FDG uptake infiltrating more than one-third of the total stomach; type II, segmental thickening of the gastric wall with elevated FDG uptake involving less than one-third of the total stomach; and type III, local thickening of the gastric wall with focal FDG uptake. The incidence of the involvement of more than one region of the stomach was higher in the patients with gastric lymphoma than in those with gastric cancer. Gastric FDG uptake was demonstrated in 23 of the 24 patients (95.8%) with gastric lymphoma and in 40 of the 43 patients (93.0%) with gastric cancer. Gastric lymphoma predominantly presented with type I and II lesions, whereas gastric cancer mainly presented with type II and III lesions. The maximal thickness was larger and the maximal standard uptake value (SUV_max_) was higher in the patients with gastric lymphoma compared with those with gastric cancer. A positive correlation between the maximal thickness and SUV_max_ was confirmed for the gastric cancer lesions, but not for the gastric lymphoma lesions. There was no difference in the maximal thickness and SUV_max_ of the gastric wall lesions between the patients without and with extragastric involvement, for gastric lymphoma and gastric cancer. Overall, certain differences exist in the findings between gastric lymphoma and gastric cancer patients on ^18^F-FDG PET/CT images, which may contribute to the identification of gastric lymphoma.

## Introduction

The gastrointestinal tract (GIT) is the most common extranodal site for non-Hodgkin’s lymphoma (NHL). Overall, 4–20% of NHLs and 30–40% of extranodal cases arise from the GIT, of which the stomach is the most frequently involved organ, followed by the small intestine, colon, pancreas and liver (1). Gastric lymphoma, secondary only to gastric cancer, has a relatively low incidence of malignant tumors of the stomach. Diffuse large B-cell lymphoma (DLBCL) and mucosa-associated lymphoid tissue (MALT) lymphoma are the two most common histological subtypes of gastric lymphoma, and other conditions, including follicular lymphoma, Burkitt’s lymphoma and T-cell lymphoma, mainly constitute the remaining subtypes (2,3).

^18^F-fluorodeoxyglucose positron emission tomography/computed tomography (^18^F-FDG PET/CT) is widely used for the diagnosis, staging, treatment response evaluation, restaging and post-therapeutic surveillance of numerous malignant tumors. By assessing the morphological changes and the metabolic status, PET/CT provides additional information to conventional imaging techniques. Numerous studies have reported the usefulness of PET or PET/CT in the management of gastric lymphoma with various histological subtypes (2–14). Endoscopic examination and direct biopsy, which provides the final diagnosis, is the established method for the identification of gastric lymphoma (15). Accordingly, ^18^F-FDG PET/CT does not have more advantages in the diagnosis of gastric lymphoma compared with endoscopy (16). Evidently, doctors who are charged with the treatment of malignant lymphoma of the stomach, request ^18^F-FDG PET/CT in order to find unanticipated lesions outside the stomach, to monitor the therapeutic response and to diagnose relapse as early as possible (12). However, unlike gastric cancer, gastric lymphoma is a group of submucosal diseases, which may be missed by gastroscopy if it occurs without destroying the mucosa (15). At this time, ^18^F-FDG PET/CT results can indicate to gastroenterologists whether a further biopsy is necessary. Furthermore, for those patients unable to undergo endoscopic examination, ^18^F-FDG PET/CT should be of clinical significance in the diagnosis of gastric lymphoma. Therefore, it is necessary to deepen our understanding of the features of ^18^F-FDG PET/CT observed in gastric lymphoma patients.

The purpose of the present study was to demonstrate the ^18^F-FDG PET/CT results of 24 patients with gastric lymphoma and to characterize the imaging features, which were compared with those of 43 patients with gastric cancer. Thus far, there has been no study of the differences in the ^18^F-FDG PET/CT results between patients with gastric lymphoma and gastric cancer.

## Patients and methods

### Patient characteristics

This retrospective study was approved by the Institutional Review Board of Jinling Hospital, School of Medicine, Nanjing University (Nanjing, Jiangsu, China) and written informed consent forms were obtained from all patients. Between August 2004 and August 2013, 24 patients who had been histologically diagnosed with gastric lymphoma by endoscopic biopsy in Jinling Hospital were reviewed retrospectively. All the patients (Table I) underwent an ^18^F-FDG PET/CT scan prior to treatment. As a comparison, 43 gastric cancer patients who underwent ^18^F-FDG PET/CT examination prior to treatment during the same time range were included in this study. The diagnoses of the gastric cancer patients (Table II) were confirmed by endoscopic biopsy or surgical specimen.

### ^18^F-FDG PET/CT imaging

All patients fasted for at least 6 h prior to receiving an intravenous injection of ^18^F-FDG (~3.7 MBq/kg body weight). Blood glucose was measured prior to the administration of ^18^F-FDG to ensure that levels were <140 mg/dl. Patients were kept lying comfortably for an uptake period of 60 min following the injection. Immediately prior to undergoing PET/CT examination, the patients drank 600 ml water to distend the stomach and were encouraged to void to minimize activity in the bladder. Scanning from the base of the skull through to the mid thigh was carried out using a PET/CT system (Biography Sensation 16; Siemens, Knoxville, TN, USA). The initial CT acquisition was performed with 120 kV, 140 mA and a slice thickness of 5 mm. The PET emission scan, with an acquisition time of 3 min for each bed, was performed immediately following CT acquisition. PET data were obtained in three-dimensional mode, with attenuation correction calculated from coregistered CT images. PET images were reconstructed using an iterative algorithm. Consequently, PET images, CT images and fused data of the two modalities were displayed on a Windows NT-based computer system (Microsoft, Redmond, WA, USA) with a Siemens/Syngo (Siemens AG, Munich, Germany) user interface.

### ^18^F-FDG PET/CT image interpretation

The ^18^F-FDG PET/CT images were visually interpreted by a consensus of two experienced nuclear medicine physicians blinded to the histological diagnosis of the patients. The images were assessed for the localization, infiltrative extent and size of lesions in the stomach as well as the presence, pattern and intensity of gastric FDG uptake. The description of the localization included specific terms representing various regions of the stomach, consisting of the cardia, fundus, body and antrum. The sizes of the lesions were recorded by measuring the maximal thickness of the gastric wall. Gastric FDG uptake was defined as increased if it was higher than the hepatic uptake or as normal if it was similar or less. If FDG accumulation occurred in the stomach, the pattern of gastric FDG uptake was classified as one of three types according to the infiltrative extent of the lesions: Type I, diffuse thickening of the gastric wall with increased FDG uptake infiltrating more than one-third of the total stomach; type II, segmental thickening of the gastric wall with elevated FDG uptake involving less than one-third of the total stomach; and type III, local thickening of the gastric wall with focal FDG uptake. Furthermore, the FDG uptake intensity of the lesions in the stomach was determined by semi-quantitatively measuring the maximal standard uptake value (SUV_max_). In addition, the presence or absence of lymph node and distant organ metastasis associated with the two malignant tumors in the stomach was also evaluated on ^18^F-FDG PET/CT images.

### Statistical analysis

The data are expressed as the mean ± standard deviation. Student’s t-test and the χ^2^ test were used to analyze statistical differences in size, SUV_max_ and categorical data between gastric lesions with lymphoma and cancer. For the size and SUV_max_ of the lesions in the stomach, Pearson’s correlation coefficient test was performed to determine the correlation. The statistical analysis was performed using SPSS 17.0 (SPSS, Inc., Chicago, IL, USA) and P<0.05 was considered to indicate a statistically significant difference.

## Results

In the 24 gastric lymphoma patients, the cardia was involved in 3 patients (12.5%), the fundus in 10 (41.7%), the body in 20 (83.3%), the antrum in 16 (66.7%) and ≥2 regions of the stomach were involved in 18 patients (75.0%). In the 43 gastric cancer patients, the incidence of the involved regions of the stomach, from the cardia to the antrum, was 39.5% (17/43), 4.7% (2/43), 39.5% (17/43) and 37.2% (16/43), respectively. The infiltrative extent of the lesion covered more than one region in only 9 of the 43 gastric cancer patients (20.9%). The incidence of cardia involvement was significantly lower (χ^2^=5.376; P<0.05) in the patients with gastric lymphoma compared with those with gastric cancer, but the incidence of the involvement of other regions, including the fundus (χ^2^=11.947; P<0.05), body (χ^2^=11.949; P<0.05) and antrum (χ^2^=5.357; P<0.05), as well as the localization larger than one region (χ^2^=18.717; P<0.001) was significantly higher.

**Figure 1:**
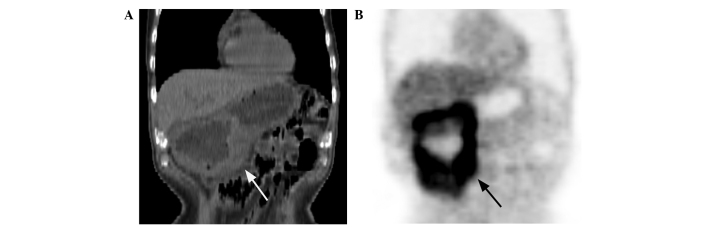
A 78-year-old male with gastric diffuse large B-cell lymphoma. (A) In the coronal computed tomography image, a diffuse thickened gastric wall can be observed in the body and the antrum of the stomach. (B) In the coronal positron emission tomography image, diffuse increased ^18^F-fluorodeoxyglucose accumulation (maximal standard uptake value, 11.3) can be confirmed in the corresponding gastric regions.

**Figure 2:**
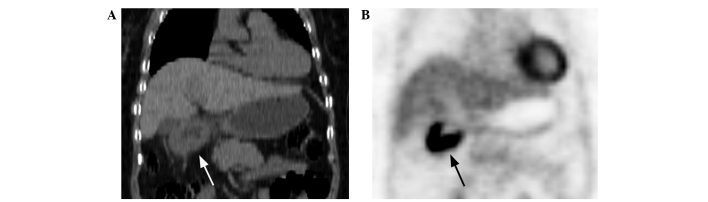
A 79-year-old female with gastric mucosa-associated lymphoid tissue lymphoma. (A) In the coronal computed tomography image, a segmental thickened gastric wall can be observed in the antrum of the stomach. (B) In the coronal positron emission tomography image, segmental increased ^18^F-fluorodeoxyglucose accumulation (maximal standard uptake value, 11.6) can be confirmed in the corresponding gastric region.

**Figure 3:**
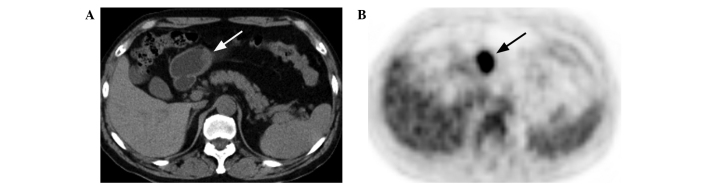
A 60-year-old male with gastric diffuse large B-cell lymphoma. (A) In the axial computed tomography image, a local thickened gastric wall can be observed in the antrum of the stomach. (B) In the axial positron emission tomography image, focal ^18^F-fluorodeoxyglucose uptake (maximal standard uptake value, 7) can be confirmed in the corresponding gastric region.

**Figure 4:**
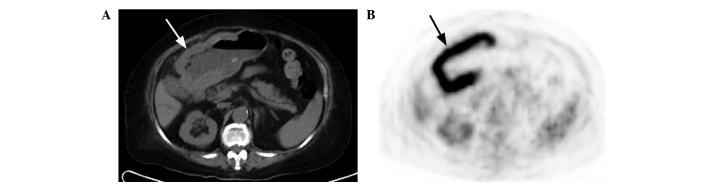
A 81-year-old female with gastric poorly-differentiated adenocarcinoma. (A) In the axial computed tomography image, a diffuse thickened gastric wall can be observed in the body and the antrum of the stomach. (B) In the axial positron emission tomography image, diffuse increased ^18^F-fluorodeoxyglucose accumulation (maximal standard uptake value, 18.5) can be confirmed in the corresponding gastric regions.

**Figure 5:**
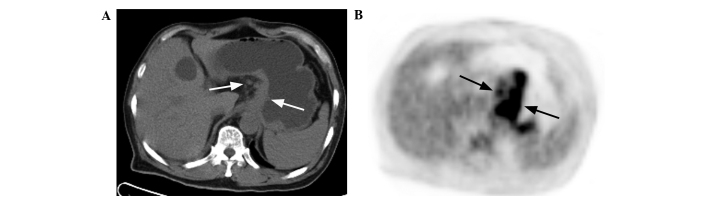
A 69-year-old male with gastric poorly-differentiated adenocarcinoma. (A) In the axial computed tomography image, a segmental thickened gastric wall can be observed in the cardia and fundus of the stomach. Enlarged perigastric lymph nodes are also apparent in this image. (B) In the axial positron emission tomography image, segmental increased FDG accumulation (maximal standard uptake value, 8.1) can be confirmed in the corresponding gastric regions. Perigastric lymph nodes with increased FDG uptake can be also observed in this image. FDG, ^18^F-fluorodeoxyglucose.

**Figure 6:**
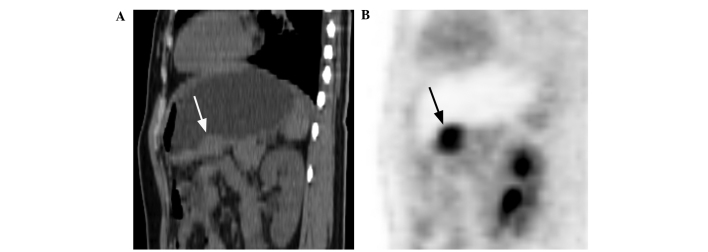
A 44-year-old female with gastric poorly-differentiated adenocarcinoma accompanied by partial signet ring cell carcinoma. (A) In the sagittal computed tomography image, local thickened gastric wall can be observed in the body of the stomach. (B) In the sagittal positron emission tomography image, local increased ^18^F-fluorodeoxyglucose accumulation (maximal standard uptake value, 5.6) can be confirmed in the corresponding gastric region.

Gastric FDG uptake was demonstrated in 23 of the 24 patients (95.8%) with gastric lymphoma and in 40 of the 43 patients (93.0%) with gastric cancer. Of the four patients with negative FDG uptake in the stomach, one case was of MALT lymphoma, one case was of moderately-differentiated adenocarcinoma and two cases were of moderately- to poorly-differentiated adenocarcinoma. With regard to the ^18^F-FDG PET/CT pattern of lesions in the stomach, type I lesions were present in 11 (47.8%; Fig. 1A and B), type II lesions in 10 (43.5%; Fig. 2A and B) and type III lesions in 2 (8.7%; Fig. 3A and B) of the 23 lymphoma patients. Type I lesions were present in 6 (15.0%; Fig. 4A and B), type II lesions in 21 (52.5%; Fig. 5A and B) and type III lesions in 13 (32.5%; Fig. 6A and B) of the 40 cancer patients. The incidence of type I lesions was significantly higher (χ^2^=7.987; P<0.01), but the incidence of type III lesions was significantly lower (χ^2^=4.562; P<0.05) in patients with gastric lymphoma when compared with the gastric cancer patients. No significant difference was identified in the incidence of type II lesions between the two groups of patients (χ^2^=0.476; P>0.05).

**Figure 7:**
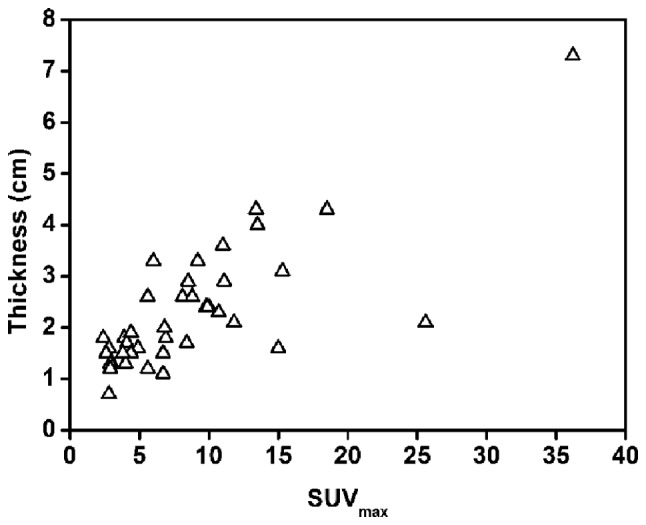
Correlation between the maximal standard uptake value (SUV_max_) and the maximal thickness for gastric cancer lesions.

**Figure 8:**
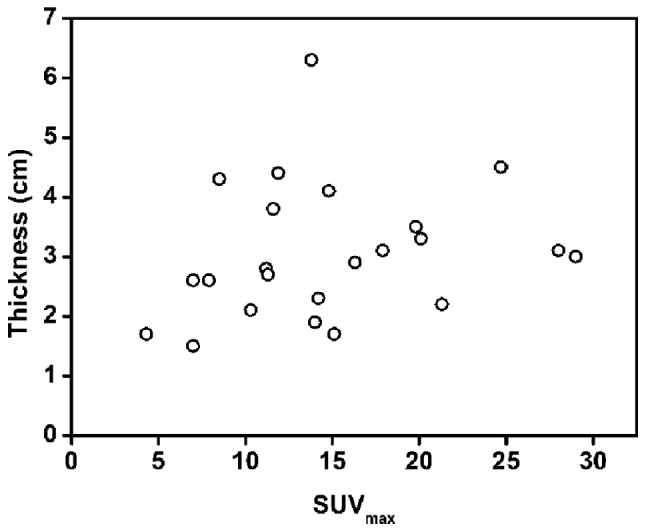
Correlation between the maximal standard uptake value (SUV_max_) and the maximal thickness for gastric lymphoma lesions.

The maximal thickness and SUV_max_ of the gastric wall lesions in the patients with gastric lymphoma and gastric cancer are compared in Table III. The maximal thickness was larger and the SUV_max_ was higher in the patients with gastric lymphoma compared with those with gastric cancer (P<0.05). In examining the association between SUV_max_ and the maximal thickness, a strong positive correlation was confirmed for the gastric cancer lesions (r=0.779, P<0.01; Fig. 7), but not for the gastric lymphoma lesions (r=0.213, P>0.05; Fig. 8).

**Figure 9:**
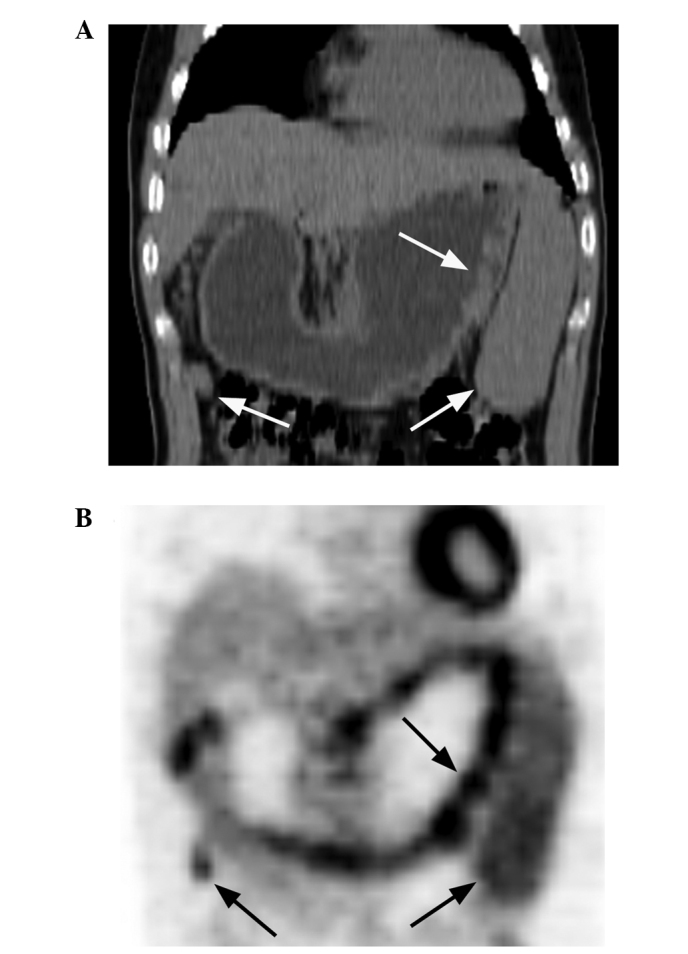
A 48-year-old male with gastric mucosa-associated lymphoid tissue lymphoma. (A) In the coronal computed tomography image, a diffuse thickened gastric wall with multiple nodules can be observed in the fundus, body and antrum of the stomach. Enlarged perigastric lymph nodes and spleen can are also apparent in this image. (B) In the coronal positron emission tomography image, a string-of-beads pattern of high FDG uptake (maximal standard uptake value, 7.9) can be confirmed in the corresponding gastric regions. Perigastric lymph nodes and spleen with increased FDG uptake are also apparent in this image. FDG, ^18^F-fluorodeoxyglucose.

The maximal thickness and SUV_max_ of the gastric wall lesions in the lymphoma patients without and with extragastric involvement (Fig. 9A and B) are compared in Table IV. The same comparisons between the cancer patients without and with extragastric involvement (Fig. 5A and B) are shown in Table V. None of these comparisons identified a statistically significant difference.

## Discussion

Gastric lymphomas are relatively rare, accounting for <5% of gastric neoplasms. Certain morphological imaging techniques have been routinely used in the processes of diagnosis, including barium X-ray and CT. However, these traditional imaging techniques have certain limitations, which may lead to no structural abnormalities being revealed. Although ^67^Gallium (^67^Ga) scans as a functional imaging modality have played an important role in diagnosing lymphoma patients, it is known to be much less sensitive in the identification of infradiaphragmatic lesions owing to physiological hepatic and splenic uptake and excretion into the bowel (17). Furthermore, there have been several studies indicating that ^67^Ga uptake in the stomach is not specific for NHL and is just as likely to occur in adenocarcinoma, gastritis and even in a normal stomach (4,17). The advantages of ^18^F-FDG PET/CT compared with conventional imaging modalities have been reported in numerous studies (11,18–20). Whether these structural and metabolic changes deriving from ^18^F-FDG PET/CT contribute to non-invasively identify gastric lymphoma requires further study.

As expected, DLBCL and MALT lymphoma accounted for the majority (23/24) of gastric lymphoma subtypes in the present study. With regard to the localization of lesions in the stomach, the cardia was less involved and the fundus, body and antrum were more involved in the gastric lymphoma patients than in the gastric cancer patients. In addition, the incidence of the involvement of more than one region of the stomach in the gastric lymphoma patients was higher than that of the gastric cancer patients. These results suggest that gastric lymphoma is inclined to infiltrate the larger extent of the gastric wall, while gastric cancer is more locally involved.

In the present study, the incidence of gastric FDG uptake was 95.8% (23/24) in patients with gastric lymphoma and 93.0% (40/43) in patients with gastric cancer. Of the 24 gastric lymphoma patients, the single patient with negative gastric tracer accumulation presented with MALT lymphoma. There are numerous PET or PET/CT studies regarding gastric MALT lymphoma, as the stomach is the most commonly involved organ in this disease (20). However, the revealed results have not been completely consistent. Enomoto *et al* (20) reported the cases of five patients with gastric MALT lymphoma, none of which exhibited abnormal tracer accumulation. Perry *et al* (21) and Radan *et al* (2) reported that gastric FDG avidity was present in only 38.9 and 71% of gastric MALT lymphoma patients, respectively. According to the studies of Ambrosini *et al* (8) and Song *et al* (6), all cases of gastric MALT demonstrated pathological FDG uptake, but the degree of FDG uptake in MALT lymphoma was much less intense in comparison to aggressive gastric NHL and was associated with therapy response. Explanations have been made for these discrepancies, including the presence of a heterogeneous cellular population (2), the shape of the lesions (20) and the physiological change or inflammatory process mocking uptake of this lymphoma type (21). Furthermore, as an indolent tumor strongly associated with *Helicobacter pylori* infection, gastric MALT lymphoma may not only exist in combination with DLBCL, but also transform into DLBCL during the follow-up period (6,19). DLBCL has been confirmed to exhibit greater accumulation of FDG than other types of lymphoma (20). Consequently, for gastric MALT lymphoma patients with a high level of uptake in the stomach, the possibility that biopsy samples did not include the large-cell portion should be considered (21). Compared with other imaging modalities, and even endoscopic biopsy, ^18^F-FDG PET/CT can minimize the misdiagnosis of DLBCL as MALT lymphoma and monitor the transformation from MALT lymphoma to DLBCL, due to the advantages of evaluating the metabolic and structural statuses of the stomach collectively (20,21).

In the current study, three gastric cancer patients without pathological trace accumulation presented with moderately-differentiated adenocarcinoma and moderately- to poorly-differentiated adenocarcinoma. By contrast, a few patients with mucinous adenocarcinoma and signet ring cell adenocarcinoma exhibited increased FDG uptake in the stomach. These results are not in agreement with several previous studies (22–26) indicating that mucinous and signet ring cell adenocarcinoma and poorly-differentiated adenocarcinoma commonly exhibit significantly low metabolic rates of FDG uptake. This disagreement is possibly due to the cause of the different stages of these gastric lesions detected by ^18^F-FDG PET/CT.

The ^18^F-FDG PET/CT pattern of gastric lymphoma has been mentioned in few previous studies (2,27). In one study (2), a diffuse or focal uptake pattern was defined according to FDG distribution in the stomach. This previous study included 55 gastric lymphoma patients, of which 30 patients (54.5%) exhibited the diffuse uptake pattern and 25 patients (45.5%) exhibited the focal pattern. The present study classified the PET/CT pattern of gastric lesions into three types according to infiltrative extent and FDG distribution in the stomach. For the 23 gastric lymphoma patients, type I (47.8%) and II (43.5%) lesions accounted for the majority, whereas type III lesions, representing focal involvement in the stomach, only accounted for the minority (8.7%). These findings are different from that of the aforementioned previous study. Additionally, for the 40 gastric cancer patients with FDG uptake in the current study, the incidence of type I, II and III lesions was 15, 52.5 and 32.5%, respectively. When the incidences were compared between patients with gastric lymphoma and those with gastric cancer, there was a statistically significant difference in type I and III lesions between the two groups, but not in type II lesions. These data indicate that the type I and II lesion ^18^F-FDG PET/CT patterns are more frequently present in gastric lymphoma patients, whereas type II and III lesions are more frequently exhibited in gastric cancer patients. Additionally, one case of gastric MALT lymphoma revealed a multiple nodular thickened gastric wall, similar to a gyrus, with a string-of-beads pattern of high FDG uptake (Fig. 9A and B), which was particular to type I lesions. This may be a novel type of MALT lymphoma that is more characteristically similar to gastric lymphoma, which requires future investigations, including a larger patient population, to elucidate.

In the present study, the maximal thickness and SUV_max_ of the gastric wall lesions were also compared between the gastric lymphoma and gastric cancer patients. The results indicated that the maximal thickness is significantly larger and that the SUV_max_ is significantly higher in patients with gastric lymphoma compared with those with gastric cancer. Consequently, the more thickened gastric wall and the higher SUV_max_ suggest that gastric lymphoma is more likely. Furthermore, gastric lymphoma on ^18^F-FDG PET/CT images should be differentiated from not only gastric cancer, but also other gastric conditions, including gastric stromal tumor, Ménétrier’s disease and even normal physiological uptake. Gastric stromal tumors are rare and usually present with an exophytic localized mass accompanied by necrosis and a well-defined margin (28). The majority of gastric stromal tumors with negative FDG uptake are benign. If the tumors exhibit high FDG uptake, they should be regarded as having malignant potential (29,30). Malignant gastric stromal tumors usually metastasize to the liver or peritoneum, but lymph node metastasis is uncommon (28,31). Ménétrier’s disease is an extremely rare disorder that is characterized by significant hypertrophy of the gastric mucosa resembling convolutions of the brain, which is accompanied by hypoproteinemia caused by the loss of proteins from the gastric mucosa. Intense ^18^F-FDG accumulation, similar to that in type I lesions, has been reported in Ménétrier’s disease in a previous study (32). However, the gastric wall thickening associated with Ménétrier’s disease tends to be most pronounced on or along the greater curvature, unlike that associated with lymphoma, which usually affects the distal stomach and lesser curvature. Furthermore, splenomegaly or lymph node enlargement may provide additional clues in diagnosing gastric lymphoma (33). Normal physiological gastric FDG uptake with a diffuse or focal pattern is not rare in clinical practice. This condition is not commonly accompanied by gastric wall thickening and can be identified by delayed PET/CT imaging with the administration of water or food (27).

Controversy remains with regard to the association between tumor size and the corresponding SUV. Certain studies have revealed a positive correlation between the two parameters in pulmonary lesions (34,35). Conversely, no significant correlation has been found between the two parameters in other tumors, including hepatic epithelioid hemangioendothelioma or ovarian metastatic tumors (36,37). Notably, the present study revealed a positive correlation between the SUV_max_ and maximal thickness in gastric cancer, but not in gastric lymphoma. The data suggested that FDG uptake in gastric cancer may be predominantly based on the tumor size, while that of gastric lymphoma may be determined by tumor size and other factors. In addition, the present study revealed that there was no difference in the maximal thickness and SUV_max_ of gastric wall lesions between patients without and with extragastric involvement, not only for gastric lymphoma, but also for gastric cancer. This may reflect the complexity of tumor invasion and metastasis, which could include multiple factors and not simply be associated with size and metabolism.

In conclusion, gastric lymphoma tends to involve more than one region of the stomach and rarely involves the cardia when compared with gastric cancer. ^18^F-FDG PET/CT has a high sensitivity in detecting gastric lymphoma and gastric cancer. In addition, gastric lymphoma predominantly presents with type I and II lesions, whereas gastric cancer mainly presents with type II and III lesions on PET/CT images. When the measurement data of PET/CT are used in identifying gastric lymphoma, a more thickened gastric wall and a higher SUV_max_ suggest that gastric lymphoma is more likely. Furthermore, gastric lymphoma and gastric cancer possess complexity regarding invasion and metastasis, which could include several factors other than tumor size and metabolism. However, the present study was limited by the small number of subjects, particularly the gastric lymphoma patients. Additionally, no cut-off SUV_max_ for identifying gastric lymphoma was confirmed. In the future, more studies with a larger number of patients will be required.

## Figures and Tables

**Table I:** Characteristics of the gastric lymphoma patients.

Characteristic	Value
Total number of patients	24
Median age, years (range)	58 (14–79)
Number of males/females	17/7
Histological subtype, n	
DLBCL	18
MALT lymphoma	5
NK/T-cell lymphoma	1

DLBL, diffuse large B-cell lymphoma; MALT, mucosa-associated lymphoid tissue; NK, natural killer.

**Table II:** Characteristics of the gastric cancer patients.

Characteristic	Value
Total number of patients	43
Median (range) age, years	69 (42–89)
Number of males/females	34/9
Histological subtype, n	
Moderately-differentiated squamous carcinoma	1
Well-differentiated adenocarcinoma	3
Well- to moderately-differentiated adenocarcinoma	2
Moderately-differentiated adenocarcinoma	13
Moderately- to poorly-differentiated adenocarcinoma	6
Poorly-differentiated adenocarcinoma	16
Accompanied by partial mucinous adenocarcinoma	1
Accompanied by partial signet ring cell carcinoma	3
Mucinous adenocarcinoma	1
Signet ring cell carcinoma	1

**Table III:** Comparisons of the maximal thickness and SUV_max_ of gastric wall lesions.

	n	Maximal thickness, cm	t	P-value	SUV_max_	t	P-value
GL	23	3.06±1.13	2.46	0.017	14.78±6.63	3.499	0.001
GC	40	2.30±1.20			8.70±6.65		

GL, gastric lymphoma; GC, gastric cancer; SUV_max_, maximal standard uptake value.

**Table IV:** Comparisons of the maximal thickness and SUV_max_ of gastric wall lesions between lymphoma patients without and with EI.

GL	n	Maximal thickness, cm	t	P-value	SUV_max_	t	P-value
No EI	6	2.85±1.17	0.522	0.607	12.53±7.25	0.965	0.345
EI	17	3.14±1.15			15.58±6.43		

GL, gastric lymphoma; EI, extragastric involvement; SUV_max_, maximal standard uptake value.

**Table V:** Comparisons of the maximal thickness and SUV_max_ of gastric wall lesions between cancer patients without and with EI.

GC	n	Maximal thickness, cm	t	P-value	SUV_max_	t	P-value
No EI	19	1.97±0.79	1.711	0.095	8.03±5.73	0.600	0.552
EI	21	2.60±1.44			9.30±7.48		

GC, gastric cancer; EI, extragastric involvement; SUV_max_, maximal standard uptake value.
